# High Trophic Niche Overlap between a Native and Invasive Mink Does Not Drive Trophic Displacement of the Native Mink during an Invasion Process

**DOI:** 10.3390/ani10081387

**Published:** 2020-08-10

**Authors:** Karla García, Carola Sanpera, Lluís Jover, Santiago Palazón, Joaquim Gosálbez, Konrad Górski, Yolanda Melero

**Affiliations:** 1 Department of Evolutionary Biology, Ecology and Environmental Sciences, University of Barcelona, 08028 Barcelona, Spain; csanpera@ub.edu (C.S.); santiago.palazon@gencat.cat (S.P.); jgosalbez@ub.edu (J.G.); 2 Facultad de Ciencias y Centro de Investigación en Biodiversidad y Ambientes Sustentables (CIBAS), Universidad Católica de la Santísima Concepción, Concepción 403000, Chile; 3 Fauna and Flora Service, Department of Territory and Sustainability, Government of Catalonia, 08017 Barcelona, Spain; 4 Instituto de Ciencias Marinas y Limnológicas, Facultad de Ciencias, Universidad Austral de Chile, Valdivia 5090000, Chile; konrad.gorski@uach.cl; 5 CREAF, 08193 Cerdanyola del Vallès, Spain; y.melero@creaf.uab.cat

**Keywords:** trophic niche overlap, stable isotopes, individual variability, invasive species, competition, *Neovison vison*, *Mustela lutreola*

## Abstract

**Simple Summary:**

Invasive species are widely recognized to negatively affect native species through both direct and indirect interactions. When diet overlap between the native and invasive species increases, their competitive interaction is expected to increase too. This in turn may lead to displacement of one of the species. However, the specific mechanisms of the diet displacement are still unclear. In this study, we analysed the diet and diet overlap between the critically endangered European mink and the invasive American mink during the invasion process of the latter species by means of stable isotope analyses. We found a significant diet overlap between the native and invasive mink when they co-occur, an important individual variation of diet, and no significant change of diet of the native species in response to the arrival of the invasive mink. These results suggest significant competitive pressure imposed on the native European mink by the invasive American mink. As such, urgent implementation of control measures of invasive species is needed to ensure the viability and conservation of endangered European mink populations.

**Abstract:**

The pressure elicited by invasive species on native species significantly increases with the increase of the overlap of their ecological niches. Still, the specific mechanisms of the trophic displacement of native species during the invasion process are unclear. The effects of the invasive American mink (*Neovison vison*) on the critically endangered European mink (*Mustela lutreola*) was assessed by analyses of diet and niche overlap during the invasion process. To do this, the isotopic composition (δ^13^C and δ^15^N) of both species of mink and their four main types of prey was analysed. Significant trophic overlap between the native European mink and invasive American mink was found when they coexisted in sympatry. Furthermore, both mink species were characterised by significant individual variation in diet and no obvious change in diet of the native species in response to the arrival of the introduced species was observed. High niche overlap registered between both species in sympatry with no displacement in diet of the native mink in response to the arrival of the invasive mink is expected to have important consequences for the viability and conservation of the native mink populations, as it suggests high competitive pressure.

## 1. Introduction

The negative effect of many invasive non-native species (INNS) on native species through direct and indirect interactions is widely recognised [1,2,3]. The pressure employed by INNS on native species is especially significant when they exercise a competitive or predator–prey interaction. As such, competitive interaction is expected to significantly increase with an increase in niche overlap between the native species and the INNS [1,3]. When species feed on different prey or have different proportions of the same prey in their diets (i.e., trophic niche partitioning occurs), trophic competition is low and the negative impact of the INNS on its native competitors may be reduced [4,5]. However, when high levels of trophic niche overlap are observed (frequently as a result of limitation of food resources available to the native species by the INNS), competitive exclusion of one of the species may occur [6]. This may then cause the native species to feed on suboptimal prey [1], eventually affecting fitness of its individuals and consequently its populations and their survival [7,8].

Despite multiple studies on trophic niche overlap between INNS and native species (e.g., [1,3,9,10,11]), little is known about the specific mechanisms of the trophic displacement that can occur between the species, most commonly to the detriment of the native species. Neither it is known if/how the diet of the native species changes during the arrival and the range expansion of the INNS. In the first stages of invasion, when INNS populations are at low densities, displacement of native competitors by the INNS due to trophic niche overlap might be low i.e., the diet of the native populations might not show any remarkable differences compared to the diet before the arrival of the INNS. However, as the INNS population grow and expand, trophic competition between the native and INNS populations is expected to increase and, as a consequence, their diets may change frequently, downgrading native species to feed on less optimal prey. Alternatively, if no change in diet occurs, two scenarios are expected: (i) native and INNS have very different niches before they enter into contact (minimal expected impacts of competition) or (ii) native and INNS have a large niche overlap and non-displacement (maximal expected impacts of competition).

After establishment, the INNS can cause a significant reduction in the trophic position (i.e., ecological role in food web) of the native populations [11,12]. Furthermore, the response of the native species to invasion depends critically on the abundance and the trophic position of the INNS that invades relative to the abundance and the trophic position of the native species [12]. Specifically, an invader with a higher trophic position compared to the native species (e.g., predators) is expected to cause a strong, nonlinear decline in the abundance of the native species’ populations, with a significant impact occurring already at a low abundance of the invader. In contrast, invaders with the same trophic position as the native species (i.e., competitors) tend to cause a linear decline in the abundance of the native species’ populations, while invaders at lower trophic levels compared to the native species’ populations are expected not to cause consistent impacts.

The American mink (*Neovison vison*, AM hereafter) is among the most widely distributed INNS and is recognised as the main threat to native European fauna [13]. Meanwhile, the European mink (*Mustela lutreola*, EM hereafter) is considered one of the most threatened mammal species in Europe [14]. Both species share most of their ecological traits and require equivalent ecological resources [15]. They have nearly identical morphologies, as well as similar habitat and trophic requirements [16,17]. Research based on scat analyses suggests that (1) the invasive mink causes variations in the diet and a potential trophic displacement of the native mink; and (2) high competitive interaction between the two species is likely to occur [16,17]. Indeed, spatial competition and the consequent distributional displacement of the EM by the AM has previously been reported [18]. In Spain, the diets of both mink species show relatively high prey diversity [19,20,21]. The EM diet is mostly based on small mammals, fish, and birds [19,20], while the AM diet contains more aquatic birds and crayfish [21]. However, the application of robust methods such as stable isotope analyses (SIA) is necessary to assess the level of trophic niche overlap and interspecific differences in the use of food resources between allopatric and sympatric populations of both mink species. Such an assessment would provide essential information to further understand the effect of the invasive mink on native fauna as well as refine approaches for control campaigns.

SIA have become increasingly common in trophic ecology studies, including diet reconstruction (e.g., [22]), assessment of spatial and temporal variation of diet (e.g., [23,24]), characterisation of trophic niches [25,26], and examination of behavioural and ecological changes [27]. The use of isotopic signatures (δ^13^C and δ^15^N) allows determination of the integrated diet consumed and assimilated over an extended period of time [28,29]. As such, the isotopic signatures of the nitrogen (δ^15^N) of the consumers indicate the trophic position of the species in the ecosystem [30,31]. Variations in the isotopic signatures of carbon (δ^13^C) provide information on the main source of the carbon used (e.g., distinguish between populations feeding on aquatic or terrestrial prey) [24,30]. Trophic niche overlap in competing populations can also be evaluated between INNS and native species using SIA (e.g., [1,3,32]). Here, we used SIA to evaluate changes in the diet of the endangered EM in relation to the arrival and expansion of the invasive AM in Spain. We studied populations before the invasion (i.e., allopatric populations) and post-invasion (i.e., sympatric populations). Specifically, we addressed the following research questions: (1) Did the diet composition of the native species change during the invasion, i.e., did their diets differ between allopatric and sympatric populations in terms of the trophic position and proportion of the prey types consumed? (2) What is the level of trophic niche overlap between the native and invasive species when they coexist in sympatry?

## 2. Materials and Methods

### 2.1. Study Area

**Figure 1:**
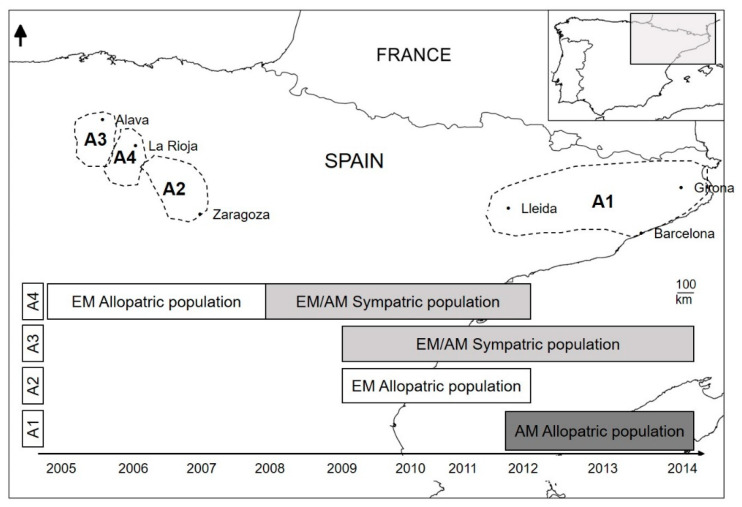
Study area showing the zones of sympatry between the American mink (AM) and the European mink (EM) populations in Spain. Area 1 (A1), AM allopatric populations; A2, EM allopatric populations; and A3 and A4, AM/EM sympatric populations. Diagram below shows the period during which each area was studied.

The study was carried out in north-eastern and northern Spain (Catalonia and La Rioja regions) over ten years (2005–2014). The spatial and temporal distributions of the EM and AM show a gradient from allopatric to sympatric populations. As part of the EM conservation project [33], both species have been recorded in the distributional area of the EM in Spain, providing information on the EM before and after the arrival and expansion of the AM (i.e., allopatric and sympatric populations). Furthermore, allopatric populations of the AM have been recorded in several areas where the EM is absent. The AM has been present in Catalonia since 1974 and in La Rioja since 2005, while the EM is only present in La Rioja. For this study, the distribution of both species was divided into four areas according to their distinct coexistence patterns: Area 1 and Area 2 included AM and EM allopatric populations, respectively; Area 3 included sympatric populations of both species; and Area 4 included the EM population before and after the arrival of the AM (i.e., allopatric and sympatric populations, Figure 1).

Among the potential prey available for the AM in Catalonia (Area 1), the most common are small rodents (the house mouse *Mus musculus* and the wood mouse *Apodemus sylvaticus* that inhabit the riparian forest), fish (cyprinids and salmonids), and the introduced American crayfish *Procambarus clarkii*. In La Rioja (Areas 2, 3, and 4), the most common prey available for the EM and the AM are fish (cyprinid, cobitids, and salmonids) as well as American crayfish and wood mice. In both regions, riparian vegetation is also the habitat for several species of waterfowls and passerines that can be preyed upon by EM and AM.

### 2.2. Sample Collection

Hair samples were collected from the carcasses of culled AM and from (non-culled) captured EM as part of the AM control and EM conservation projects [33]. The AM and EM samples were released by the Spanish Ministry of Agriculture, Food and Environment and were provided by technicians from regional governments. Both mink species were captured in wire mesh traps (60 × 15 × 15 cm), as authorised by the autonomous authorities. Each EM specimen was marked with a subcutaneous passive transponder and released once the individual had fully recovered from anaesthesia, whilst all AM specimens that were captured were subsequently transferred to official wildlife rehabilitation centres for euthanasia following the Spanish Animal Welfare Law (Royal Act No. 32/2007).

Altogether, 44 EM samples (23 females and 21 males) and 85 AM samples (30 females and 55 males) were obtained. All EM samples were collected in autumn, while all AM samples were collected during both autumn and winter. Samples from both species were grouped according to the area of capture location and in relation to their coexistence pattern (allopatric or sympatric). As such, the following number of samples was collected for each of the coexistence combinations: 25 EM samples from individuals in allopatry (Area 2 and 4; from 2005 to 2012), 19 EM samples from individuals in sympatry with AM (Area 3 and 4; 2008–2012), 46 AM samples from individuals in allopatry (Area 1), and 39 AM samples from individuals in sympatry with the EM (Area 3) (2012–2014, Figure 1). Samples from potential prey based on published diet data (i.e., small mammals, birds, fish, and crayfish) were also collected [19,20,21]. Traps, nets, and hand capture were used to collect the animals over a 2–3 day period in Catalonia and La Rioja during 2014 and 2015. Isotopic signatures were analysed in muscle tissue from small mammals, fish, crayfish, and bird feathers.

### 2.3. Stable Isotope Analyses

Stable isotope analyses were conducted on mink hair samples. Prior to the analyses, all hair samples were rinsed in a NaOH (0.25 M) solution, washed in distilled water, and dried in a stove at 60 °C. Subsequently, samples were homogenised with scissors. Approximately 0.4 mg of hair was weighed in tin buckets (3.3 × 5 mm) and combusted at 900 °C. Analyses of the prey samples were conducted on 1 to 10 individuals of each prey species (Table S1). Muscle tissue from each specimen was subjected to lipid extraction by successive rinsing in a chloroform and methanol solution (2:1), following the protocol of Folch et al. [34], to minimise the differences in δ^13^C caused by the variable concentrations of tissue lipids [35]. All prey samples (muscle tissue from mammals, fish, crayfish, and bird feathers) were oven-dried at 60 °C and mechanically ground into a fine powder. Subsequently, approximately 0.3 mg of each sample was weighed in tin buckets (3.3 × 5 mm) and combusted at 900 °C. Nitrogen and carbon isotopic analyses were carried out by isotope ratio mass spectrometry (EA-IRMS) using a Thermo-Finnigan Flash EA 1112 analyser coupled to a Delta isotope ratio mass spectrometer via a ConFlo III interface. The stable isotope signatures of carbon and nitrogen (δ^13^C and δ^15^N)were expressed in parts per thousand (‰) and were calculated with the following equation:

$$\delta X\left(‰\right)=\left[\left(\frac{R_{sample}}{R_{standard}}\right)-1\right]$$

where X is ^15^N or ^13^C and *R* the corresponding ratio ^15^N/^14^N or ^13^C/^12^C. The δ^13^C standard was Vienna PeeDee Belemnite (VPDB) calcium carbonate and the δ^15^N standard was atmospheric nitrogen (N_2_). International standards (ammonium sulphate, potassium nitrate, and glutamic acid for δ^15^N; and polyethylene, sucrose, and glutamic acid for δ^13^C) were used after every 12 samples to calibrate the equipment and compensate for any drift over time. The precision and accuracy were ≤0.1‰ for δ^13^C and ≤0.3‰ for δ^15^N.

### 2.4. Data Analyses

Values of the stable isotope ratios of carbon and nitrogen for both mink species were checked for normal distribution using Q–Q plots, while Levene’s test was used to assess homogeneity of the variances. Since both carbon and nitrogen ratio values showed a normal distribution, parametric tests were conducted. To test the differences in diet of the EM between allopatric and sympatric populations, an ANOVA was performed. We used δ^13^C and δ^15^N as the dependent variables and the coexistence pattern (i.e., allopatric and sympatric) and sex as the explanatory variables, while year was set as a random effect. An initial model considering all the potential independent variables and their first order interactions was constructed. A backward stepwise procedure was used to obtain the final model. A similar procedure was used to test for differences between the sexes in the diet of allopatric AM populations. Thus, an ANOVA was performed using δ^13^C and δ^15^N as the dependent variables and sex as the explanatory variable. All statistical analyses were performed using IBM SPSS Statistics for Windows version 23 (IBM Corp., Armonk, NY, USA).

To test for changes in the composition of the diet in terms of the type and proportion of prey consumed (i.e., mammals, birds, fish, and crayfish) in allopatric and sympatric populations, a Bayesian mixing model was used. Mixing models estimate the probability distributions of the multi-source contributions to a consumer diet by accounting for variation and uncertainties in the input data. The shift in isotope ratios between the food sources and consumer tissue was incorporated by adding trophic discrimination factors (TDFs). Given that there is no literature data on the trophic discrimination in mink and we did not evaluate the TDFs experimentally, we decided to use the TDFs presented by Newsome et al. [36,37] in the sea otter, *Enhydra lutris nereis*, vibrissae—3.5‰ for δ^15^N and 2.5‰ for δ^13^C. The mixing model analysis was undertaken using the SIAR library in R [38]. Subsequently, we estimated the trophic position (TP) of the EM in R using the Bayesian package tRophicPosition [39] and the *Onebaseline* model. The tRophicPosition model includes isotopic variation in the baseline indicator, the consumer, and the trophic discrimination in order to provide a robust estimation of consumer TP at the population level.

To test the degree of trophic niche overlap between the two mink species in co-existence, we first quantified the trophic niche width of both mink species using the total convex hull area encompassed by all individuals (TA) [40]. We calculated the isotopic niche breadths of both mink species using the Stable Isotope Bayesian Ellipses (SIBER) model [41] in the SIAR package. The standard ellipse area (SEAc) provides a measure of the isotopic niche of each individual [40,41], and was used to assess the isotopic niche overlap between two species. The SEAc contains around 40% of the isotopic data, thereby representing the core isotopic niche of each individual while correcting for variable sample sizes [40,41,42]. Additionally, a similar analysis was used for the AM to determine possible differences between the sexes in relation to the trophic niche overlap.

## 3. Results

### 3.1. European Mink Diet

**Figure 2:**
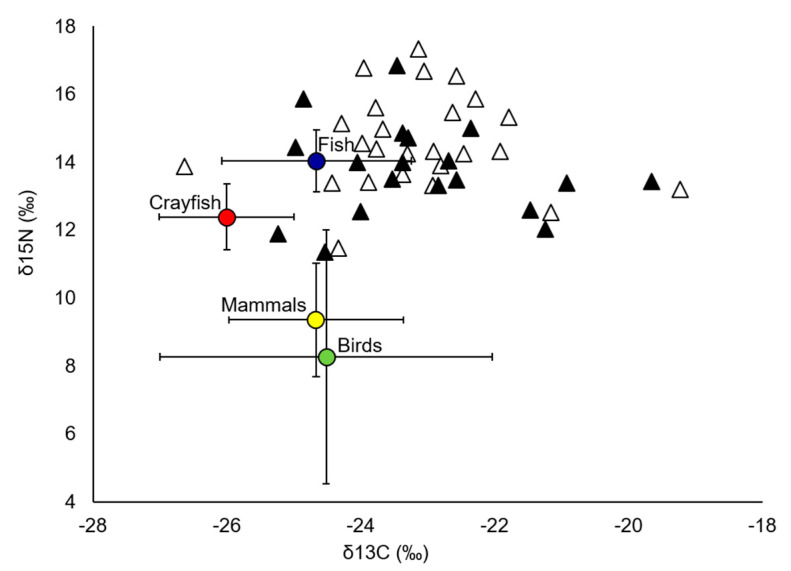
Scatterplot of the dispersion of the δ^15^N and δ^13^C isotopic signatures of hair samples from the European mink in the pre-invasion (open triangles) and post-invasion (filled triangles) stages. Prey isotopic signatures are shown as means (±SD).

A high degree of inter-individual variability in the δ^15^N (11 to 17‰) and δ^13^C (−27 to −19‰) values was observed in the allopatric and sympatric populations of EM (Figure 2). Terrestrial prey, birds (8.26 ± 3.73‰), and mammals (9.35 ± 1.67‰) showed a significantly lower mean δ^15^N compared to aquatic prey, crayfish (12.38 ± 0.97‰), and fish (14.04 ± 0.91‰) (F_1,444_ = 62.17, *p* < 0.0001).

Overall, isotopic values indicated a decrease in the consumption of prey from high trophic levels (δ^15^N, 14.6 to 13.7‰) and a slight increase in the consumption of more terrestrial prey (δ^13^C, −23.7 to −23.1‰) in sympatric compared to allopatric populations (Figure 2). However, these differences were not statistically significant (δ^15^N: F_1,7_ = 3.51, *p* = 0.07; δ^13^C: F_1,0.01_ = 0.01, *p* = 0.98). Similarly, no significant differences between the sexes were found for δ^15^N (F_1,1_ = 0.50, *p* = 0.48) and δ^13^C (F_1,3.1_ = 1.59, *p* = 0.22) between allopatric and sympatric populations (Table S2). However, the δ^13^C values varied between the years (F_7,4_ = 2.44, *p* = 0.04), while there were no differences in the δ^15^N values between years (F_7,18_ = 1.38, *p* = 0.24).

**Figure 3:**
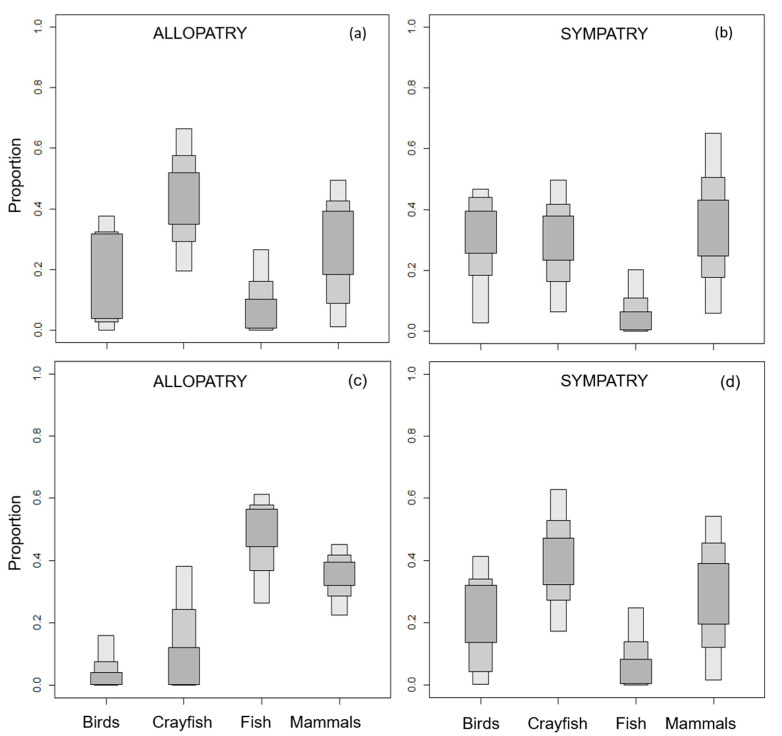
Diet compositions of the European mink in (**a**) allopatry and (**b**) sympatry, and of the American mink in (**c**) allopatry and (**d**) sympatry according to the SIAR mixing model, using the indirect vibrissae discrimination factor. The contribution of each prey to the diet is shown with 95% (light grey), 75% (medium grey), and 50% (dark grey) credibility intervals.

SIAR Bayesian mixing models revealed that crayfish (~43% of the diet) and small mammals (~26%) were the predominant prey for allopatric EM populations (Figure 3a). In contrast, EM in sympatry with AM mostly fed on small mammals (~35% of the diet), whereas the proportion of crayfish (~29%) and fish (~8%) was lower (Figure 3b).

**Figure 4:**
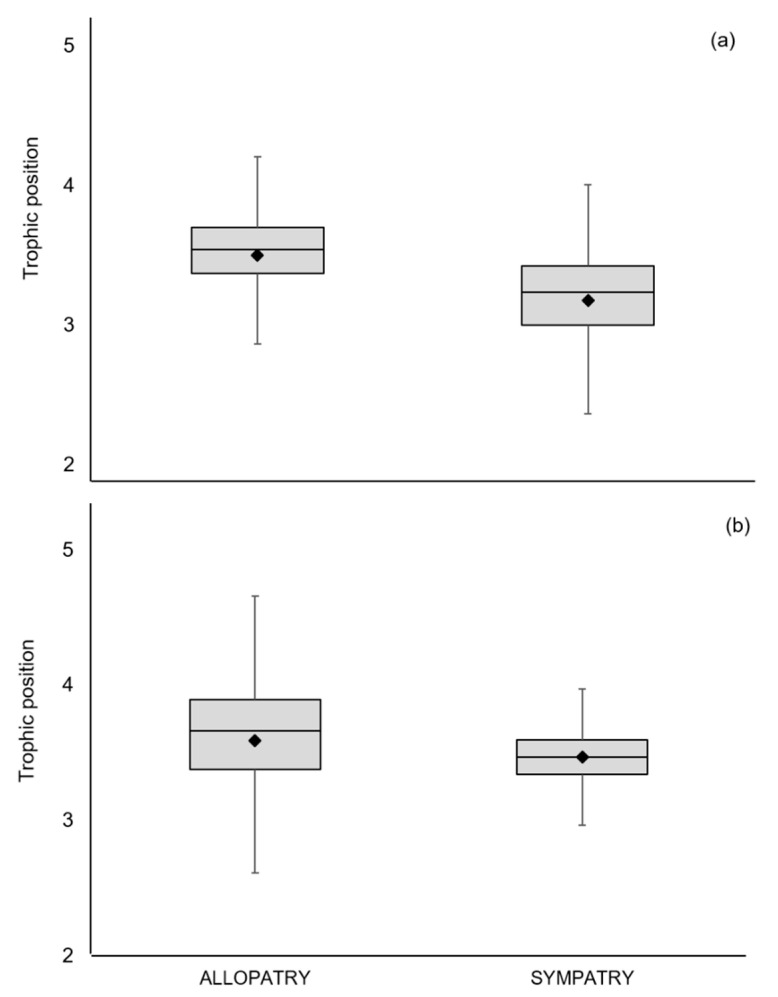
Estimates of trophic position of the (**a**) European mink and (**b**) American mink in allopatry and sympatry. Bars show the median and mean (diamond) trophic position and 95% credibility intervals, calculated relative to a baseline using tRophicPosition.

Bayesian estimates of TP supported the mixing model results, with a decrease in the consumption of prey of a higher trophic level in sympatric compared with allopatric populations. The mean (95% credibility intervals) estimated TP for the EM allopatric samples was 3.5 (2.1–4.3), while for sympatry the estimated TP decreased to 3.2 (2.0–4.0) (Table 1, Figure 4a).

### 3.2. American Mink Diet

The AM diet in areas where the species occurred in allopatry was mostly composed of fish (~45%) and small mammals (~34%, Figure 3c). However, when in sympatry with the EM, the consumption of fish and small mammals decreased, while that of crayfish and birds increased (~ 40% and ~21%, respectively; Figure 3d).

Both the δ^15^N and δ^13^C values were higher in females than males in the allopatric and sympatric populations, but these differences were not statistically significant (δ^13^C: F_1,0.02_ = 0.006, *p* = 0.937; δ^15^N: F_1,11_ = 2.393, *p* = 0.129; Table S2). The diet overlap analysis between the sexes showed a considerably higher trophic niche width (i.e., TA and SEAc) of males compared to females (TA _males_ = 45.97, TA _females_ = 14.46). SEAc showed an almost complete overlap between the standard ellipses calculated from the δ^15^N and δ^13^C values for both sexes. Specifically, the overlap in the ellipses constituted 81.59% of the female ellipse and 42.41% of the male ellipse (Figure S1).

Bayesian estimates of TP revealed a similar trophic position in sympatric and allopatric populations (Table 1, Figure 4b). The mean (95% credibility limits) estimated TP for the AM in allopatric populations was 3.6 (2.0–4.7), whereas in sympatric populations it was 3.5 (2.8–4.2).

### 3.3. Trophic Niche Width and Niche Overlap

**Figure 5:**
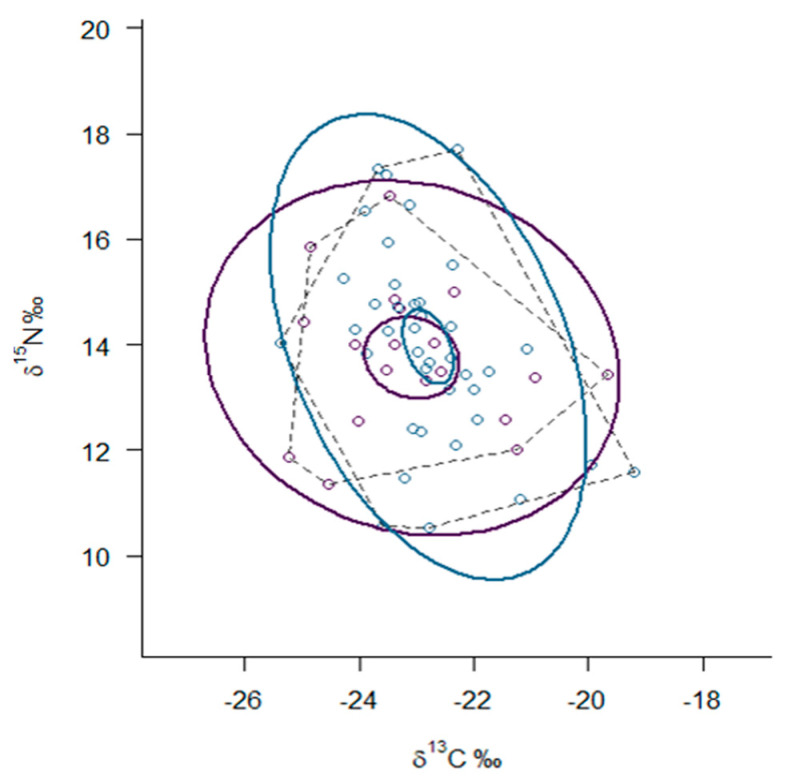
Stable isotope Bayesian ellipses (solid lines) represent the trophic niche width and overlap between the European mink (purple) and American mink (blue) based on standard ellipses corrected for small sample sizes (SEAc), with a 95% confidence interval for the means of each species. SEAc represents the core niche area of each group, while the convex hulls represent the overall niche diversity and encompass all data points. Each circle represents an individual mink.

Analyses of the stable isotopes of carbon and nitrogen in hair samples from both mink species in sympatry revealed a larger trophic niche width in the AM compared to the EM (TA_AM_ = 26.02, TA_EM_ = 18.85, Table 1), indicating a wider range of prey type consumed by the AM. SEAc showed an almost complete overlap between the standard ellipses calculated from the δ^15^N and δ^13^C values for both species, indicating high trophic overlap between the two species in sympatry (Figure 5) and suggesting that most of the surface of the ellipses is shared between the two species. Specifically, the overlap in the ellipses of the EM and AM constituted 71.73% of the EM ellipse and 80.29% of the AM ellipse. These results corroborate results obtained from the SIAR Bayesian mixing model, which showed a very similar proportion of prey types between the two species when they were in sympatry.

## 4. Discussion

Our results revealed a high trophic niche overlap in sympatric populations of the native and invasive mink, and a high individual variability in the diet of both species in allopatric and sympatric populations. Nevertheless, and unlike previous studies based on faecal analysis [16,17], no significant change in diet of the native species in response to the arrival of the introduced species was registered. A high trophic niche overlap between the species, as well as no displacement of the native mink in response to the arrival of the invasive mink, could indicate significant trophic competition between both mink species. These findings could partly explain the decline in the EM in areas colonized by the invasive AM in Spain [18]. However, next to trophic competition, as revealed by our results, other direct antagonistic interactions and take-over of territories should also be considered as significant factors causing declines in EM populations in response to AM invasion [18,43,44,45].

The invasive mink was characterised by a broader range of δ^15^N values compared to the native mink in both allopatry and sympatry. This suggests that, compared to EM, invasive AM may have a greater ability to exploit diverse food resources. Maran et al. [16] suggested that when confronted with the same spectrum of available food items and in the absence of other species, both mink species chose the same or similar food items. However, they found significant differences between the diet of EM and AM when a large spatial scale was considered. These differences were attributed to differences in habitat selection [16]. Similarly, a habitat shift in combination with reduced prey abundance has been proposed as a mechanism for the diet change of EM in response to invasion by AM [17]. Introduced non-native predators may cause a significant decrease in the abundance of native prey in their new habitat [46,47], which may increase the competitive interactions between the invasive and native predators [17]. As a result, a decline in the trophic position of the native predator may occur [9,46]. We found no evidence of this effect in the present study based on the mammal invasion model of the species, as only a slight and statistically not significant decline in the trophic position of EM coexisting in sympatry with AM was observed.

Interestingly, a high intraspecific variation (measured as the dispersion of δ^15^N and δ^13^C isotopic signatures) in the diet of both the EM and AM was observed in both allopatry and sympatry. This indicates a considerable individual variability in the feeding habits of both species, with individuals consuming prey of different trophic levels and using different sources of carbon (aquatic or terrestrial). Thus, our results confirmed the individual differences in feeding in both the native and invasive mink species, which has only been previously shown by Sidorovich et al. [48] in Belarus. As such, although both EM and AM are generalist predators, some individuals exhibit a predominantly terrestrial diet (i.e., mammals and birds), while others base their diets on aquatic resources (i.e., fish and crayfish) in both allopatry and sympatry. Furthermore, our results revealed that variability in the diet between individuals was higher than the variability between the sexes. Indeed, previous studies have documented no difference in diet between sexes for both native EM [19] and invasive AM [49]. As such, these lack of differences in the diet between sexes for both mink species suggest that both females and males have similar feeding habits regardless of whether they are in allopatry or sympatry.

Previous studies of the EM diet in Romania and Spain using scat analyses reported that aquatic prey was highly abundant in its diet in allopatry (relative frequency of occurrence of up to 30–35%) [19,50]. In Belarus, Sidorovich et al. [17] found that fish together with crayfish and especially frogs formed the main part of the EM diet before the expansion of the invasive AM. During the progression of the AM invasion, the EM trophic niche has narrowed, and the proportion of aquatic prey in its diet has declined considerably. Although our results also showed a reduction in the consumption of aquatic prey in sympatric populations, fish were the least consumed prey in both allopatric and sympatric EM populations (13% and 8%, respectively). The discordances between our and the results of previous studies could be related to methodological differences as analyses of scats or stomach content can overestimate the abundance of slowly or no digestible prey items in the diet [29]. However, these differences could also be caused by differences in prey abundance and availability in each studied area. Similarly, the differences in diet between sympatric and allopatric populations of AM populations could have been caused by differences in prey availability as these populations are geographically distant, though in Mediterranean ecosystems riparian habitats potentially provide a similar prey composition.

SIAR Bayesian mixing models provided robust evidence that the native and invasive mink consumed similar diets in sympatry, gaining most of their energy and nutrients from similar prey types. However, a narrower trophic niche width was observed for the native mink when it coexists with the invasive mink, which is concordant with the results of Maran et al. [16] and Sidorovich et al. [17]. As in other invasive species [51], this suggests that the invasive mink has greater flexibility in their habitat use and feeding habits compared to the native mink. Our results also revealed a similar trophic position for both mink species between the allopatric populations, and a decrease in the isotopic trophic position was registered in the native mink when it coexisted with the invasive mink. This indicates that when both species coexist the invasive mink is feeding at a higher trophic level (TP = 3.5) compared to the native mink (TP = 3.2).

Trophic niche overlap, suggesting dietary competition, has been confirmed previously in co-occurring native and invasive species [1,3,52,53,54]. We also observed a high trophic overlap (>70%) when both mink species coexisted in sympatry, suggesting interspecific competition. Sidorovich et al. [17] also reported high trophic overlap (50–61%) between the two mink species in sympatry in Belarus. In contrast, Maran et al. [16] reported low trophic overlap in their study of EM and AM in sympatry and suggested that both species may reduce the potential competition and facilitate coexistence by niche partitioning. The mechanisms that may allow such niche partitioning may relate to changes in diurnal activity time or distance to a water body [55]. However, the high trophic overlap observed for EM and AM coexisting in sympatry in Spain could indicate that the native EM cannot adapt their trophic requirements quickly in response to the arrival of the invasive AM. Alternatively, perhaps there is sufficient availability of terrestrial and aquatic prey to support the dietary requirements of both the invasive and native predators. However, since seasonal changes have been observed in the diet of both EM and AM [19,21], future studies should undertake stable isotope analyses during contrasting periods of the year to evaluate if the trophic niche overlap between the two species is constant or changes throughout the year.

The ecological and social impacts caused by the INNS on invaded areas have been widely recognized (e.g., [56,57,58]). However, the ultimate ecological impacts of many invasions that have occurred worldwide in recent decades are yet to be understood [56]. As an INNS, the American mink has generated a significant negative impact on the native trophic cascades as well as on local economic activities (e.g., fish farms, sport fishing, and bird watching tourism) in places where it was introduced (e.g., [58,59]). However, its trophic interactions with native species within the same guild have not shown clear patterns and varied among the study areas due to differences in the geographic and ecological settings, but also due to the assessment methodologies employed [16,17]. Our study reinforces the utility of stable isotope analyses to reveal the trophic interactions between related species as it permitted an assessment of diet integrated over larger time periods, which improved our understanding of the competitive interactions between native and invasive mink in Spain. As such, monitoring of the trophic interactions between these two mink species could be an important way to assess whether the American mink control measures applied in the study area are effective.

## 5. Conclusions

This study presents evidence for a significant trophic overlap between native European mink and invasive American mink. Furthermore, both mink species are characterised by significant individual diet variability and no significant change in the diet of the native species in response to the arrival of the introduced species was observed. High niche overlap between the native European mink and invasive American mink in sympatry with no displacement of the native mink in response to the arrival of the invasive mink might have important consequences for the viability and conservation of native mink populations, as it suggests significant competitive pressure.

## Figures and Tables

**Table 1:** The mean (±SD) δ^13^C and δ^15^N values of each mink species and their primary carbon sources. n = number of samples; TP = trophic position (95% credibility intervals); TA = Total area; SEAc = standard ellipse area corrected for a small sample size, representing the isotopic niche metrics calculated for both mink species in sympatry based on the δ^13^C and δ^15^N values.

Species/Metrics	n	δ^13^C (‰)	δ^15^N (‰)	TP	TA	SEAc
Allopatry						
*Mustela lutreola*	25	−23.13 (1.36)	14.57 (1.41)	3.5 (2.1–4.3)		
*Neovison vison*	45	−23.83 (1.72)	14.84 (2.22)	3.6 (2.0–4.7)		
Sympatry						
*Mustela lutreola*	19	−23.08 (1.48)	13.75 (1.37)	3.2 (2.0–4.0)	18.84	6.68
*Neovison vison*	40	−22.78 (1.10)	13.87 (1.84)	3.5 (2.8–4.2)	26.08	5.97
Primary sources						
Crayfish	7	−26.00 (1.01)	12.38 (0.97)			
Fish	22	−24.66 (1.42)	14.04 (0.91)			
Birds	31	−24.52 (2.48)	8.26 (3.73)			
Mammals	11	−24.67 (1.30)	9.35 (1.67)			

## Supplementary Materials

The following are available online at https://www.mdpi.com/2076-2615/10/8/1387/s1, Figure S1: Stable isotope Bayesian ellipses (solid lines) represent the trophic niche overlap between the sexes for the American mink based on standard ellipses corrected for small sample sizes (SEAc), with a 95% confidence interval for the means of each species. SEAc represents the core niche area of each group, while the convex hulls represent the overall niche diversity and encompass all data points. Each circle represents an individual mink. Males and females are represented in blue and purple, respectively. Table S1: Ratios of stable isotopes of carbon and nitrogen (mean ± standard deviation) of potential prey of the American and European mink in the two analysed regions. Table S2: Mean δ^15^N and δ^13^C values ± SD of hair samples from American and European mink in allopatric and sympatric populations.
