# Supplementary material for: High Trophic Niche Overlap between a Native and Invasive Mink Does Not Drive Trophic Displacement of the Native Mink during an Invasion Process
Source: Animals (Basel). 2020 Aug 10;10(8):1387. doi: 10.3390/ani10081387 (PMC7460352; doi:10.3390/ani10081387)
Supplement: Supplementary file 1 [file animals-10-01387-s001.pdf]

Supplementary Material

High trophic niche overlap between a native and invasive mink does not drive trophic displacement of the native mink during an invasion process

Karla P. García [1,2], Carola Sanpera [1], Lluís Jover [1,†], Santiago Palazón [1,3], Joaquim Gosálbez [1], Konrad Górski [2,4] and Yolanda Melero [5]

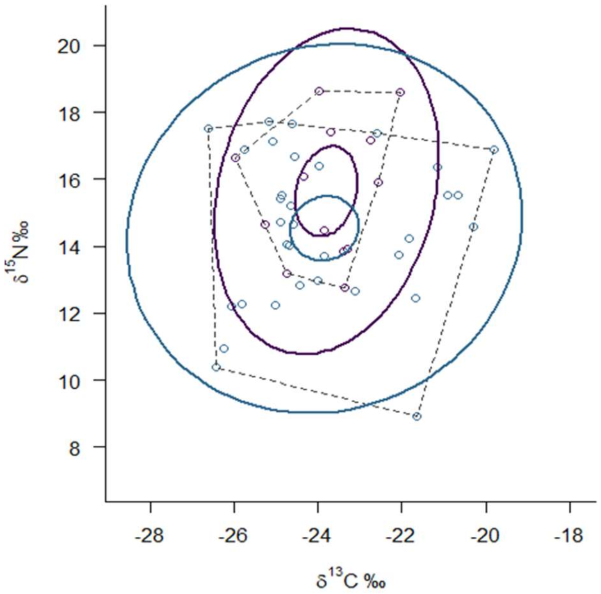

Figure S1. Stable isotope Bayesian ellipses (solid lines) represent the trophic niche overlap between the sexes for the American mink based on standard ellipses corrected for small sample sizes (SEAc), with a 95% confidence interval for the means of each species. SEAc represents the core niche area of each group, while the convex hulls represent the overall niche diversity and encompass all data points. Each circle represents an individual mink. Males and females are represented in blue and purple, respectively.

Table S1. Ratios of stable isotopes of carbon and nitrogen (mean ± standard deviation) of potential prey of the American and European mink in the two analysed regions.

**Sample**					
**Species**	**Common name**	**Family**	***n***	**$\delta^{15}N$ (mean ± SD)**	**$\delta^{13}C$ (mean ± SD)**
Catalonia					
Crustaceans					
Procambarus clarkii	American crayfish	Cambaridae	3	13.4 ± 0.6	-24.9 ± 3.6
Fish					
Barbus meridionalis	Western Mediterranean barbel	Cyprinidae	1	16.6	-29.3
Cyprinus carpio	Common carp	Cyprinidae	1	14.1	-24.1
Gobio gobio	Gudgeon	Cyprinidae	5	16.4 ± 0.3	-24.4 ± 0.9
Parachondrostoma miegii	Ebro nase	Cyprinidae	1	15.6	-24.1
Phoxinus phoxinus	Eurasian minnow	Cyprinidae	6	16.4 ± 1.5	-25.0 ± 0.6
Squalius laietanus	Ebro chub	Cyprinidae	1	14.9	-29.4
Birds					
Aegithalos caudatus	Long-tailed tit	Aegithalidae	10	10.9 ± 0.7	-24.8 ± 0.8
Cyanistes caeruleus	Eurasian blue tit	Paridae	2	6.7 ± 5.9	-22.3 ± 1.8
Erithacus rubecula	European robin	Muscicapidae	3	5.1 ± 2.8	-24.0 ± 0.9
Fringilla coelebs	Common chaffinch	Fringillidae	1	8.51	-16.56
Regulus regulus	Goldcrest	Sylviidae	1	4.73	-25.70
Sylvia melanocephala	Sardinian warbler	Sylviidae	1	10.21	-22.93
Troglodytes troglodytes	Eurasian wren	Troglodytidae	1	7.07	-26.27
Mammals					
Apodemus sylvaticus	Long-tailed field mouse	Muridae	5	6.1 ± 1.3	-24.8 ± 0.6
Mus spretus	Algerian mouse	Muridae	5	5.2 ± 1.9	-24.2 ± 1.7

La Rioja					
Crustaceans					
Procambarus clarkii	American crayfish	Cambaridae	7	12.4 ± 1.0	-26.0 ± 1.0
Fish					
Cyprinus carpio	Common carp	Cyprinidae	2	12.4 ± 0.5	-25.6 ± 0.0
Gobio gobio	Gudgeon	Cyprinidae	5	13.6 ± 0.9	-24.4 ± 0.4
Parachondrostoma miegii	Ebro nase	Cyprinidae	5	14.3 ± 0.5	-26.2 ± 0.4
Phoxinus phoxinus	Eurasian minnow	Cyprinidae	5	14.1 ± 0.5	-24.6 ± 0.4
Salmo trutta	Brown trout	Salmonidae	5	14.8 ± 0.7	-23.1 ± 1.8
Birds					
Cettia cetti	Cetti's warbler	Scotocercidae	1	15.85	-26.37
Cisticola juncidis	Zitting cisticola	Cisticolidae	1	11.23	-23.42
Cyanistes caeruleus	Eurasian blue tit	Paridae	3	3.4 ± 0.3	-24.1 ± 1.4
Emberiza schoeniclus	Reed bunting	Emberizidae	1	7.82	-16.68
Erithacus rubecula	European robin	Muscicapidae	3	4.8 ± 0.3	-25.1 ± 0.7
Miliaria calandra	Corn bunting	Emberizidae	2	7.9 ± 0.1	-22.7 ± 0.0
Panurus biarmicus	Bearded reedling	Panuridae	4	12.2 ± 1.2	-28.6 ± 1.4
Phylloscopus collybita	Common chiffchaff	Phylloscopidae	3	9.3 ± 3.7	-25.0 ± 1.2
Prunella modularis	Dunnock	Prunellidae	1	3.65	-23.22
Sylvia atricapilla	Eurasian blackcap	Sylviidae	5	6.7 ± 3.8	-24.7 ± 2.0
Sylvia melanocephala	Sardinian warbler	Sylviidae	2	11.6 ± 0.1	-23.0 ± 0.5
Turdus merula	Eurasian blackbird	Turdidae	1	12.03	-23.79
Turdus philomelos	Song thrush	Turdidae	4	8.1 ± 2.3	-23.7 ± 1.0
Mammals					
Apodemus sylvaticus	Long-tailed field mouse	Muridae	5	9.4 ± 1.8	-25.0 ± 1.5
Crocidura russula	White-toothed shrew	Soricidae	1	10.02	-24.81
Mus spretus	Algerian mouse	Muridae	5	9.2 ± 1.9	-24.3 ± 1.3

Table S2. Mean $\delta^{15}N$ and $\delta^{13}C$ values ± SD of hair samples from American and European mink in allopatric and sympatric populations.

	American mink				European mink			
	Females		Males		Females		Males	
	δ15N	δ13C	δ15N	δ13C	δ15N	δ13C	δ15N	δ13C
Allopatric	15.6 ± 1.9	-23.7 ± 1.1	14.5 ± 2.3	-23.8 ± 1.9	14.8 ± 1.6	-23.6 ± 1.2	14.3 ± 1.1	-22.6 ± 1.4
Sympatric	14.1 ± 1.7	-23.1 ± 0.6	13.9 ± 1.9	-22.6 ± 1.4	13.8 ± 1.1	-23.1 ± 1.3	13.7 ± 1.7	-23.1 ± 1.7
